# Appendage Resection and Pulmonary Vein Isolation on Minimally Invasive Coronary Artery Bypass Graft

**DOI:** 10.1016/j.atssr.2023.11.022

**Published:** 2023-12-12

**Authors:** Hiroki Sakai, Keita Kikuchi, Kaito Masuda, Yoshun Sai, Kunihiko Yoshino, Joji Ito

**Affiliations:** 1 Department of Cardiovascular Surgery, Tokyo Bay Urayasu Ichikawa Medical Center, Chiba, Japan

## Abstract

Minimally invasive coronary artery bypass grafting is becoming standardized; however, its small incision may result in a limited field of view. Challenges arise in performing left atrial appendage resection and pulmonary vein isolation from the same incision, whereas safety and feasibility are not well documented. Our report demonstrates safe achievement of left atrial appendage resection and pulmonary vein isolation from the same minimally invasive coronary artery bypass grafting wound site with a good surgical field of view. In addition, we present an intraoperative positional change technique for right-sided pulmonary vein isolation.

Minimally invasive cardiac surgery–coronary artery bypass grafting (MICS-CABG) was performed successfully more than a decade ago.^1^ With advances in minimally invasive treatments, positive outcomes have been observed in coronary artery stenosis and arrhythmia treatment with use of catheters.^2^ In addition, excellent results of surgical pulmonary vein isolation (PVI) for refractory atrial fibrillation (AF) have been reported,^3^ leading to increased demands for standardized MICS-CABG and coronary artery bypass. Here, we describe a novel technique in which CABG, left atrial appendage resection (LAAR), and PVI by a less invasive approach are successfully performed in a patient with AF.

A 54-year-old man with palpitations due to paroxysmal AF presented to our hospital 1 year before surgical procedure. Despite several catheter ablations for AF, the symptoms recurred. Coronary angiography performed during catheter ablation revealed a 90% stenosis of the left main trunk. Subsequently, the patient underwent MICS-CABG with LAAR and PVI at our hospital.

On admission, the patient was hemodynamically stable. His blood pressure was 123/80 mm Hg. The patient had no signs of heart failure. Electrocardiography confirmed a normal sinus rhythm (68 beats/min). Transthoracic echocardiography revealed no left ventricular enlargement, 65% ejection fraction of the left ventricle, and no significant valvular dysfunction.

Under general anesthesia, the patient was intubated with a double-lumen endotracheal tube for 1-lung ventilation. Two pressure bags were placed on his back to facilitate intraoperative changes in position from the left semidecubitus to the right semidecubitus position. The bag on the right side of the patient's back was pressurized, whereas that on the left side was deflated, placing the patient in a left semidecubitus position. The first step involved PVI on the right side. Four ports were inserted into the right thoracic space. The right pulmonary veins were taped, and PVI was performed with a cardiac electrosurgical device.

The bag on the left side of the patient's back was pressurized, that on the right side was deflated, and the patient was placed in the right semidecubitus position. For MICS-CABG, the surgical field was expanded as described by Kikuchi and colleagues.^4^ An 8-cm left thoracotomy was performed in the fifth left intercostal space below the left nipple. A 12-mm port was inserted in the left sixth intercostal posterior axillary line for the insertion of an automated anastomotic device for the LAAR. A stabilizer was inserted, and the right lung was compressed dorsally to secure the visual field. The bilateral internal thoracic artery was harvested with a long-shaft harmonic scalpel. Three deep pericardial sutures were stitched to the pericardium. An automated anastomotic device was inserted through the device port into the sixth left intercostal space (Figure 1A). Transesophageal echocardiography confirmed that the left arterial appendage lumen had completely collapsed, and the left atrial appendage was resected by an automated anastomotic device (Figure 1B). The resected edge of the left atrial appendage was reinforced with an end-loop polydioxanone (PDS Ⅱ) suture. The Marshall ligament was cut, and the left pulmonary veins were dissected and taped. Left PVI was performed with a cardiac electrosurgical device, as was right PVI (Figure 1C). The left internal thoracic artery was anastomosed to the left anterior descending coronary artery and the right internal thoracic artery to the obtuse marginal branch and high lateral branch with 8-0 polypropylene. The operation time was 249 minutes. Postoperative coronary computed tomography showed patency of all the bypass grafts (Figure 2).

**Figure 1:**
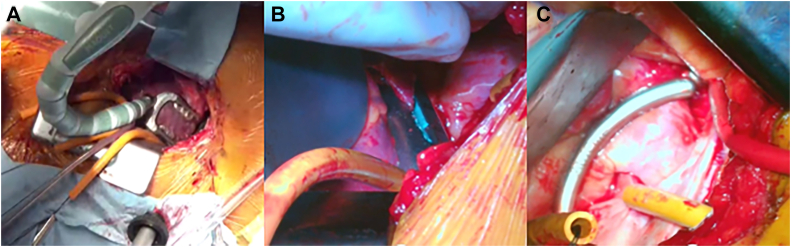
(A) Insertion of device from sixth intercostal space. (B) Left atrial appendage resection view. Insertion of the device through the left sixth intercostal space allows complete left atrial appendage resection without difficulty. (C) Left pulmonary vein isolation view. The cardiac electrosurgical device is inserted from the left sixth intercostal space, and left pulmonary vein isolation is performed.

**Figure 2:**
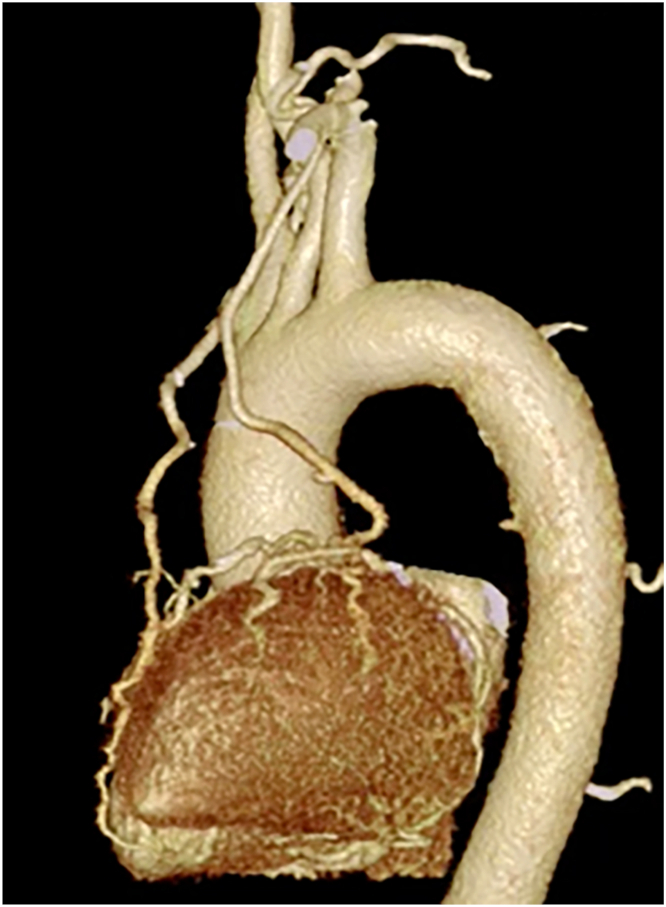
All coronary bypasses are patent.

## Comment

Since the first report of MICS-CABG, favorable outcomes have been documented in several studies.^1^^,^^5^ MICS-CABG preserves the sternum, which not only facilitates smooth postoperative recovery of the patient but also prevents sternitis and mediastinitis associated with median sternotomy in high-risk patients, such as patients with diabetes and receiving dialysis. This study describes successful off-pump MICS-CABG using bilateral in situ internal thoracic arteries and video-assisted bilateral PVI and LAAR, thereby expanding the possibility of MICS-CABG regardless of AF status, preserving its sternum-sparing benefits.

The procedure's major challenge arises from the need for different patient positions during MICS-CABG and right PVI. However, we successfully achieved the ideal position using 2 pressurized bags on the patient's back, adjusting them during the procedure. MICS-CABG is typically performed with an 8-cm skin incision at the fifth intercostal thoracotomy site. However, observing the left atrial appendage under off-pump conditions in the fifth intercostal thoracotomy has been challenging, which previously prevented simultaneous MICS-CABG and LAAR. Our study demonstrated that with appropriate field development and proper port positioning, MICS-CABG with video-assisted PVI and LAAR can be performed without complications.

To date, MICS-CABG, LAAR, and PVI have been considered technically challenging in CABG cases with AF. As a result, conventional CABG with full sternotomy, LAAR, and PVI have been performed instead of MICS-CABG. Catheter ablation for AF and transcatheter left atrial appendage closure, known as the Watchman procedure, are sometimes performed before or after MICS-CABG. Catheter ablation for AF has favorable outcomes,^2^ and the Watchman procedure has shown promising results in patients with AF.^6^ However, performing LAAR and PVI simultaneously by MICS-CABG would be more efficient, allowing the discontinuation of anticoagulants after surgical procedure.^7^ PVI in patients with unsuccessful catheter ablation for AF has been reported to have a higher rate of return to sinus rhythm than additional catheter ablation.^3^

Reliable LAAR with intraoperative transesophageal echocardiography is mandatory to ensure the complete stroke-preventive effect.^8^ At our hospital, a cardiologist performed intraoperative transesophageal echocardiography, ensuring a successful LAAR. In some patients, discontinuation of antithrombotic drugs for AF is necessary because of gastrointestinal bleeding or hemorrhagic complications. In this group, who are often in poor general condition, a less invasive procedure such as MICS-CABG combined with LAAR and PVI is highly demanded.

It is clear that CABG through a median sternotomy approach is still the “gold standard.” On the other hand, the use of bilateral internal thoracic arteries in diabetic patients and dialysis patients carries the risk of mediastinitis. This sternum-preserving technique may be an option to prevent mediastinitis in these groups of patients.

In conclusion, MICS-CABG with LAAR and PVI is a safe and technically feasible procedure. This novel technique expands the possibilities for surgical treatment in AF cases, offering potential benefits in managing complex patients with AF.
